# Effectiveness, Safety, and Acceptability of Primaquine Mass Drug Administration in Low-Endemicity Areas in Southern Thailand: Proof-of-Concept Study

**DOI:** 10.2196/51993

**Published:** 2024-06-26

**Authors:** Jaranit Kaewkungwal, Wanlapa Roobsoong, Saranath Lawpoolsri, Wang Nguitragool, Suwich Thammapalo, Pathomporn Prikchoo, Amnat Khamsiriwatchara, Rungrawee Pawarana, Pawinee Jarujareet, Daniel M Parker, Piyarat Sripoorote, Mondha Kengganpanich, Chetta Ngamjarus, Jetsumon Sattabongkot, Liwang Cui

**Affiliations:** 1 Department of Tropical Hygiene Mahidol University Bangkok Thailand; 2 Mahidol Vivax Research Unit Faculty of Tropical Medicine Mahidol University Bangkok Thailand; 3 Department of Molecular Tropical Medicine and Genetics Faculty of Tropical Medicine Mahidol University Bangkok Thailand; 4 Ministry of Public Health Nonthaburi Thailand; 5 Center of Excellence for Biomedical and Public Health Informatics (BIOPHICS) Faculty of Tropical Medicine Mahidol University Bangkok Thailand; 6 Department of Population Health and Disease Prevention University of California, Irvine Irvine, CA United States; 7 Department of Epidemiology and Biostatistics University of California, Irvine Irvine, CA United States; 8 Department of Health Education and Behavioral Sciences Faculty of Public Health Mahidol University Bangkok Thailand; 9 Department of Epidemiology and Biostatistics Faculty of Public Health Khon Kaen University Khon Kaen Thailand; 10 Division of Infectious Diseases and Internal Medicine Department of Internal Medicine University of South Florida Tampa, FL United States

**Keywords:** mass drug administration, cluster-crossover randomized controlled trial, community-based trial, participatory epidemiology, Plasmodium vivax, primaquine

## Abstract

**Background:**

A challenge in achieving the malaria-elimination target in the Greater Mekong Subregion, including Thailand, is the predominance of *Plasmodium vivax* malaria, which has shown extreme resilience to control measures.

**Objective:**

This proof-of-concept study aimed to provide evidence for implementing primaquine mass drug administration (pMDA) as a strategy for *P. vivax* elimination in low-endemicity settings.

**Methods:**

The study employed a mixed-methods trial to thoroughly evaluate the effectiveness, safety, acceptability, and community engagement of pMDA. The quantitative part was designed as a 2-period cluster-crossover randomized controlled trial. The intervention was pMDA augmented to the national prevention and control standards with directly observed treatment (DOT) by village health volunteers. The qualitative part employed in-depth interviews and brainstorming discussions. The study involved 7 clusters in 2 districts of 2 southern provinces in Thailand with persistently low *P. vivax* transmission. In the quantitative part, 5 cross-sectional blood surveys were conducted in both the pMDA and control groups before and 3 months after pMDA. The effectiveness of pMDA was determined by comparing the proportions of *P. vivax* infections per 1000 population between the 2 groups, with a multilevel zero-inflated negative binomial model adjusted for cluster and time as covariates and the interaction. The safety data comprised adverse events after drug administration. Thematic content analysis was used to assess the acceptability and engagement of stakeholders.

**Results:**

In the pre-pMDA period, the proportions of *P. vivax* infections in the pMDA (n=1536) and control (n=1577) groups were 13.0 (95% CI 8.2-20.4) and 12.0 (95% CI 7.5-19.1), respectively. At month 3 post-pMDA, these proportions in the pMDA (n=1430) and control (n=1420) groups were 8.4 (95% CI 4.6-15.1) and 5.6 (95% CI 2.6-11.5), respectively. No statistically significant differences were found between the groups. The number of malaria cases reduced in all clusters in both groups, and thus, the impact of pMDA was inconclusive. There were no major safety concerns. Acceptance among the study participants and public health care providers at local and national levels was high, and they believed that pMDA had boosted awareness in the community.

**Conclusions:**

pMDA was associated with high adherence, safety, and tolerability, but it may not significantly impact *P. vivax* transmission. As this was a proof-of-concept study, we decided not to scale up the intervention with larger clusters and samples. An alternative approach involving a targeted primaquine treatment strategy with primaquine and DOT is currently being implemented. We experienced success regarding effective health care workforces at point-of-care centers, effective collaborations in the community, and commitment from authorities at local and national levels. Our efforts boosted the acceptability of the malaria-elimination initiative. Community engagement is recommended to achieve elimination targets.

**Trial Registration:**

Thai Clinical Trials Registry TCTR20190806004; https://www.thaiclinicaltrials.org/show/TCTR20190806004

## Introduction

### Background

As part of the World Health Organization (WHO) campaign for “zero malaria,” the Greater Mekong Subregion (GMS) of Southeast Asia has developed a strategic plan for regional malaria elimination by 2030, while Thailand aims to achieve this goal by 2024 [1,2]. One major challenge facing the GMS is the predominance of *Plasmodium vivax* malaria [3,4], which has shown extreme resilience to control measures [5,6]. Initiatives to eliminate malaria have an excellent impact on *Plasmodium falciparum* but not on *P. vivax* worldwide due to the various unique aspects of *P. vivax* biology. One challenge is asymptomatic infections with *P. vivax*, which are undetected and untreated, potentially contributing to transmission over several weeks or months [7]. Asymptomatic infections are especially common in low-endemicity areas where control tools have reduced the malaria burden [8-10]. A study along the Thailand-Myanmar border noted that while the proportion of severe *P. vivax* malaria varied across different geographic regions and transmission settings, a significant proportion of the community had asymptomatic parasitemia, even in low-transmission areas [9].

The identification and appropriate treatment of asymptomatic infected individuals, who are typically missed by clinical case-based surveillance, have become critical for interrupting malaria transmission in the final elimination phase [11,12]. Several approaches have been proposed to accelerate *P. vivax* elimination, for example, novel serological test-and-treat interventions, radical cure strategies, case-centered surveillance and response systems, and mass drug administration (MDA) [13-15]. To deal with low blood parasitemia and the formation of hypnozoites associated with *P. vivax* infection that evade conventional diagnosis, presumptive preventive treatment of an endemic population by MDA using a hypnozoiticidal drug, such as primaquine (PQ), is often the chosen strategy to eliminate residual *P. vivax* transmission [15-17]. Large-scale MDA with pyrimethamine and PQ was associated with decreased *P. vivax* transmission in central and southern China [14].

The WHO mentioned the lessons learned from MDA implementation (so-called “mass primaquine preventive treatment [MPPT]”) in several temperate countries, for example, MPPT combined with vector control and other preventive measures resulted in the rapid containment of *P. vivax* epidemics and may have contributed to the interruption of transmission in low-transmission settings [18]. Prior to 2019, there were no data on the implementation of MPPT in tropical and subtropical areas. In 2019, when this project started in Thailand, the WHO reported 2 MDA studies in the GMS with differing results, with one study demonstrating only a short-term reduction in *P. vivax* transmission and the other study finding no effect [18]. It appears that MDA with PQ has been successful in eliminating *P. vivax* in the temperate zone [11,15,19,20], but its applicability and effectiveness for eliminating *P. vivax* in tropical countries remain to be evaluated.

Therefore, as part of the International Center of Excellence for Malaria Research (ICEMR) project, supported by the US National Institutes of Health (U19 AI089672), this study was conducted to evaluate the effectiveness, safety, and acceptability of MDA with PQ in low-endemicity areas in a tropical country. Particularly, this study was considered a proof-of-concept evaluation of MDA with PQ (termed primaquine mass drug administration [pMDA]) using a cluster-crossover randomized trial design to provide the evidence base for designing and implementing a pMDA strategy in low malaria-endemic settings in Thailand. With the planning of the pMDA strategy by the National Malaria Control Program in Thailand, we also sought to critically assess the acceptability and engagement of stakeholders at various levels. According to the main ICEMR project, if pMDA (phase I) was proven to be effective, safe, and acceptable, the pMDA intervention would be scaled up to cover over 100,000 villagers in 200 clusters in 8 provinces (phase II) using a stepped-wedge design to provide a statistically robust evaluation of pMDA.

### Objectives

The goal of this proof-of-concept study was to thoroughly assess the effectiveness, safety, acceptability, and community engagement of pMDA as a strategy for malaria elimination in Thailand. The specific aims were as follows: (1) to compare the proportions of *P. vivax* infections between communities under the national standard of prevention and control (SPC) program and communities under the SPC program augmented with pMDA, (2) to assess the safety of PQ implemented in the communities, and (3) to assess the acceptability and stakeholder engagement of the pMDA intervention for possible scale-up to phase II.

## Methods

### Trial Design

The study employed a mixed-method design comprising quantitative and qualitative approaches. The quantitative part, which was used for assessing the effectiveness and safety of the pMDA intervention, adopted a cluster-crossover randomized controlled design, a modified cluster randomized design, which is particularly feasible in pragmatic clinical trials in health care systems [21,22]. This design was selected as it is suitable for determining the effectiveness of a routinely used intervention in health care practice, in which the intervention is randomized (turned on and off) at the community level instead of the individual level [23]. In this study, clusters were randomized to a sequence of treatment conditions. A group of clusters received pMDA in the first period (year 1) and the SPC program in the second period (year 2), while the other group of clusters received these preventive activities in reverse order.

The qualitative part, which was used for assessing the acceptability and stakeholder engagement of the pMDA intervention, used in-depth interviews (IDIs) and brainstorming sessions among stakeholders. Stakeholders were primarily sensitized before the study started and informed regularly during the study period.

### Settings and Locations

**Figure 1:**
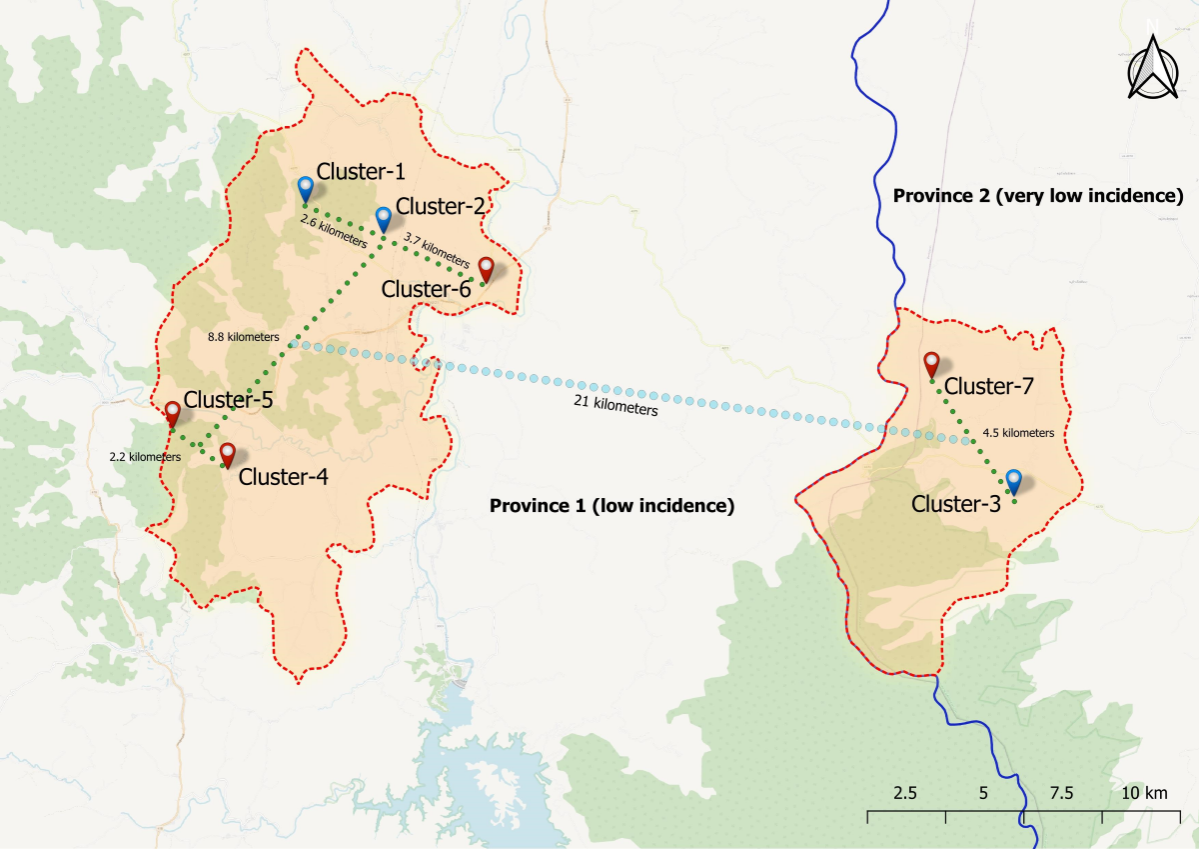
Clusters assigned to the primaquine mass drug administration (pMDA) group vs the control group in Yala and Narathiwat provinces during the 2-period cluster-crossover randomized controlled trial.

The study was conducted in 2 districts of 2 southern provinces in Thailand (Yala and Narathiwat), which had persistently low *P. vivax* malaria transmission. The study sites comprised 7 purposively selected villages, the smallest administrative unit in Thailand, with each typically having about 200-400 residents. The 7 villages were selected according to their reported malaria incidences in 2018 from routine passive case detection in the malaria surveillance system of Thailand (unpublished data). Five villages (#1, 2, 4, 5, and 6) were in Yala Province, with *P. vivax* incidence rates of 4%-7%, and 2 had unexpectedly high rates (over 30%) in 2018. Two villages (#3 and 7) were in Narathiwat Province, with *P. vivax* incidence rates of 1%-2%. For the study design, one group of villages served as the pMDA treatment group, while the other served as the control group. In the following year, the intervention was swapped between the 2 groups of villages. A map showing locations and distances among the 2 sets of 7 clusters (clusters 1-3 vs clusters 4-7) in the 2 provinces is presented in Figure 1. The nearest distances between 2 sets of clusters were 3.7-4.5 km. It should be noted that primary health care management in each village was independent. On average, a village health volunteer (VHV) is responsible for providing health care services to 10 households in the village. Each VHV assists the local health workers in promoting health, preventing diseases, and providing basic health services to local communities. In this study, VHVs were assigned to implement pMDA and collect data from the study participants in the nonoverlapping households for which they were responsible.

### Study Participants

For the quantitative study, all villagers living in the selected clusters were invited to join the study if they were aged 1 year or older. For PQ administration, the exclusion criteria were as follows: (1) pregnancy and lactation, (2) age <7 years, (3) glucose-6-phosphate dehydrogenase (G6PD) deficiency, (4) hemoglobin level <8 g/dL, (5) body weight <15 kg, (6) history of allergy to PQ, and (7) history of drug reactions, such as hemolysis or dark urine, after PQ intake.

For the qualitative study, key informants included stakeholders representing the following 4 levels of engagement and participation: national level (malaria experts or consultants, Thailand Ministry of Public Health [MOPH]), regional and provincial level (health officers or authorities), district level (personnel at operational units of district hospitals), and local level (community leaders, religion gatekeepers, and VHVs). Villagers in the community were also invited, regardless of whether they participated in pMDA activities.

### Intervention

The control group was exposed to the SPC program, a routine malaria prevention and control program, implemented by the MOPH. The SPC program includes routine case reports, case investigations, and disease- or vector-control activities at the village level. Besides routine activities, eligible subjects within the pMDA group received a dose of 0.25 mg/kg of PQ daily for 14 days. The 0.25 mg/kg dose was selected according to the WHO recommendation and evidence supported by previous studies, as it is well tolerated in G6PD-normal individuals [11,24]. Those who were G6PD deficient were excluded from PQ administration. Directly observed treatment (DOT) was performed to ensure compliance.

### Outcomes and Study Procedures

**Figure 2:**
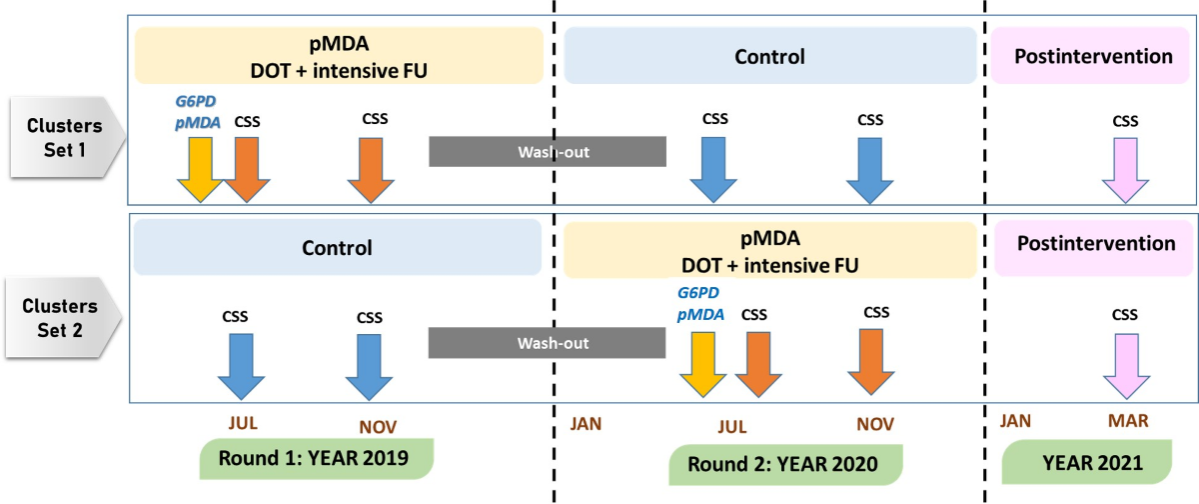
Study procedures to assess the effect of primaquine mass drug administration (pMDA) for reducing *P. vivax* infection in the community in a 2-period cluster-crossover randomized controlled trial during the period 2019-2022. CSS: cross-sectional blood survey; DOT: directly observed treatment; FU: follow-up; G6PD: glucose-6-phosphate dehydrogenase.

For the quantitative study, the primary outcome was the proportion of *P. vivax* infections among the study participants within each cluster before and after 2 rounds of pMDA. The secondary outcome was the safety of the study participants who took PQ, which was closely monitored during the 14-day treatment. The study procedure is shown in Figure 2. Baseline demographic information was collected using a structured questionnaire at the start of the study. One-time testing for G6PD was performed for all villagers, using the qualitative CareStart G6PD rapid diagnostic test, before pMDA to assess the eligibility of study participants to receive PQ. For each round of pMDA, an initial cross-sectional blood survey (CSS) was conducted in both the pMDA and control groups before pMDA implementation, and a follow-up CSS was performed 3 months after pMDA. As an additional postintervention follow-up, a CSS was performed for both groups 6 months after the second round of pMDA. At each survey, finger-prick blood was collected from each participant to prepare dried filter-paper blood spots, which were later used for *Plasmodium* detection by quantitative polymerase chain reaction (qPCR) to identify asymptomatic infection cases in the CSS population. At the time of each survey period, those in the CSS population who had clinical symptoms and were detected by the microscopic method in the routine MOPH malaria surveillance system were also subsequently verified by qPCR and counted as confirmed *P. vivax*–infected cases. The prevalence, defined as the proportion of confirmed *P. vivax* infections at the community level in different periods, was calculated from the number of PCR-confirmed *P. vivax* cases (both asymptomatic and symptomatic cases) divided by the total number of cases in each round of the CSS.

For the qualitative study, the primary outcome was information about the acceptability and stakeholder engagement of the pMDA intervention implemented in the localities. Information was collected using IDIs at the study participants’ homes and community meeting places. A brainstorming meeting at the regional health office was also arranged for collective opinions among representative authorities at the national, regional, and provincial levels. Data collection was performed after round 2 at the conclusion of the study.

### Sample Size

For the quantitative study, the sample size was calculated with the notation that it was a proof-of-concept study to assess the potential impact of pMDA implementation. A go or no-go decision to scale up the study with a larger sample size would be made based on the potential effectiveness and feasibility of the intervention according to the interclass cluster correlation effect obtained from this study. We planned for a sample size of 1500 study participants per group. With this sample size, the power to detect differences between the 2 groups varied according to the baseline proportions of *P. vivax* in the study areas, with a 2-tailed type I error of 5%. With a sample size of 1500 per group, when the baseline proportion of *P. vivax* in the cluster is low at 3% (30 in 1000) and the effect sizes (difference between the pMDA and control groups) are 50% and 75%, powers of 79.1% and 99.5%, respectively, would be achieved. In areas with a very low baseline at 1% (10 in 1000) and effect sizes of 50% and 75%, the study would have detection powers of 35.5% and 74.1%, respectively.

For the qualitative study, IDIs were planned to include study participants from 24 families and at least six key informants from 2 villages of each province, including those who completed PQ administration, those who did not complete pMDA, and those who did not participate in pMDA. IDIs were planned for stakeholders in pMDA areas, including 12 VHVs (at least six per province), 4 health care authorities (district and provincial levels), and 4 local leaders or gatekeepers (village heads and religious masters). A brainstorming meeting at the conclusion of the study was planned with 15 representatives, including MOPH consultative experts, authorities from regional and district vector-borne units, authorities from regional and district health offices, staff from hospitals at the subdistrict level, and staff who worked in the selected areas.

### Randomization

To plan and evaluate the intervention, we subdivided the selected study areas into villages, which are the smallest administrative units in Thailand. A village is considered a cluster and treated as the unit of randomization. The sizes of the purposively selected clusters by authorities in the study areas were about 150-250, with a larger size of around 600 residents. Considering the cluster sizes and the distances between clusters, we purposively allocated 2 out of 5 villages in Yala and 1 out of 2 villages in Narathiwat to receive the pMDA intervention in round 1 (year 1) and switch to the control group in round 2 (year 2).

### Statistical Methods

The potential effectiveness of pMDA was explored in terms of reductions in the prevalences and proportions of confirmed *P. vivax* infections on qPCR in the CSS population, with a comparison between the intervention and control groups. This was a 2-period cluster-crossover study involving 2 cluster periods and multiple CSSs before and after pMDA in each period. As suggested in the literature, the data should be analyzed with hierarchical models with random effects in order to allow for different outcome probabilities in each period, cluster, and cluster period [25,26]. With a cluster-level randomization design, it is statistically more efficient to employ a model adjusted for the cluster effect [22]. As a proof-of-concept for a pragmatic trial in the community setting, the potential effectiveness of pMDA was thus determined by comparing the proportions of *P. vivax* infections between the 2 groups at each period of CSS, with the 2 periods combined representing the overall impact of the intervention. An additional comparison of the proportions of *P. vivax* infections between the 2 groups at 6 months after round 2 was also performed to explore the longer-term effect. As the study was conducted in low-endemic areas, it is more likely that most study participants would not be infected. Therefore, the analysis used the R package NBZIMM, which provides functions for setting up a multilevel zero-inflated negative binomial model adjusted for random intercepts of the clusters [27,28]. The model for the overall impact of the intervention in the 2-period crossover was adjusted for the cluster effect plus time period and the interaction between the time period and intervention. The prevalence ratio and its 95% CI from the model have been reported, and statistical significance was set at *P*<.05.

The safety of the pMDA strategy was monitored in terms of adverse events that occurred after drug administration throughout the follow-up period. All events have been presented descriptively. The prevalence of G6PD deficiency has been described with its 95% CI.

The acceptability and stakeholder engagement of the pMDA strategy were examined using qualitative analysis. IDIs and brainstorming sessions were led by trained facilitators, including an experienced lead facilitator, 4 co-facilitators and note-takers, and 2 local health care staff. All team members had been trained on ethical considerations for human research subjects and had read the study protocol and data collection methodologies. They discussed the important points to be explored. Audio recordings of the IDIs and meetings were transcribed, and the transcripts were reviewed and classified into key themes according to the study objectives and themes that emerged during the reviews. Thematic analyses were conducted on the notes of participant responses and the determined themes. The content was analyzed to identify themes by manually exploring, interpreting, and categorizing the data via consensus among facilitators. The priority themes set according to the study objectives included the following: perceptions, expectations, engagement, factors influencing the decision to participate or not to participate, factors influencing noncompletion of the 14-day regimen, perceived achievements, blockage and solutions in pMDA, and challenges in pMDA implementation.

### Ethical Considerations

This study was approved by the Ethics Committee of the Faculty of Tropical Medicine, Mahidol University, Bangkok, Thailand (approval number: MUTM 2019-033-01). All participants consented to participate in this study before enrollment. Participants younger than 18 years were consented or assented along with consent from their parents. Community leaders and authorities in national and local health facilities were informed. Moreover, they provided consent and were involved as part of the community sensitization prior to the study initiation. All staff responsible for research activities were trained in human subject research protection. The identifiable information of study participants was treated as confidential information, and the participants’ identification numbers were coded. To treat pMDA as an additional part of the routine malaria prevention activities by health care personnel and VHVs in natural settings, no compensation was provided to the consented study participants. Generative AI was not used in any portion of manuscript preparation.

## Results

### Study Participant Characteristics and Intervention Implementation and Coverage

A total of 2550 individuals resided in 7 clusters. In round 1, 1624 participants consented to the CSS, with 925 in clusters 1-3 and 699 in clusters 4-7 being allocated to the pMDA and control groups, respectively. In the 3 villages allocated to pMDA, 70.8% (655/925) of individuals were eligible according to the inclusion and exclusion criteria, and 87.8% (575/655) received pMDA. The follow-up CSS at 3 months after PQ administration was performed among 1423 study participants, with 92.0% (851/925) in the pMDA clusters versus 81.8% (572/699) in the control clusters.

**Figure 3:**
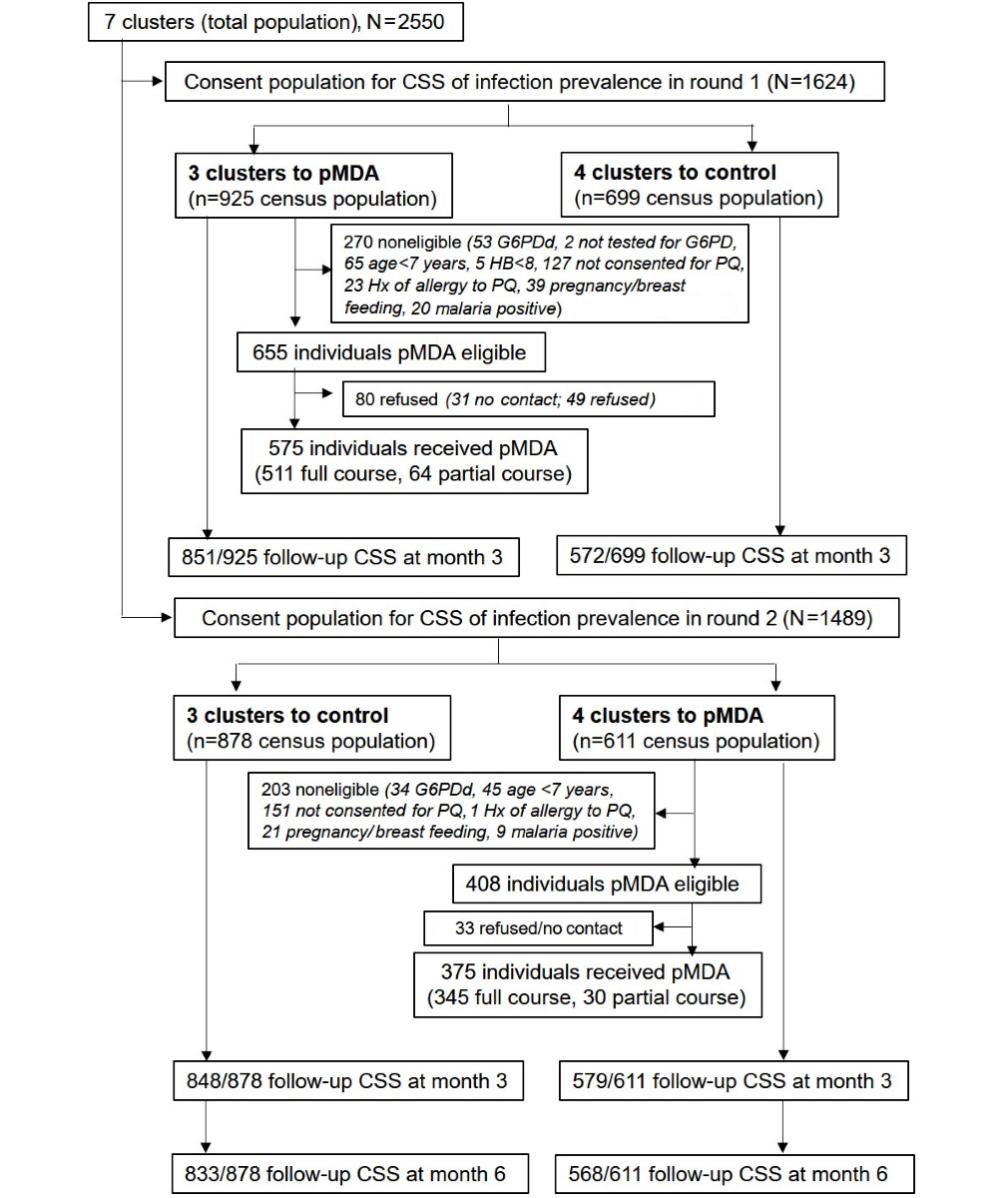
CONSORT (Consolidated Standards of Reporting Trials) diagram showing the flow of participants throughout the 2-period cluster-crossover trial. CSS: cross-sectional blood survey; G6PD: glucose-6-phosphate dehydrogenase; G6PDd: glucose-6-phosphate dehydrogenase deficiency; HB: hemoglobin; Hx: history; pMDA: primaquine mass drug administration; PQ: primaquine.

In round 2, the clusters were crossed over, and 1489 participants consented to the CSS, with 878 in clusters 1-3 and 611 in clusters 4-7 being switched to the control and pMDA groups, respectively. In the 4 villages that switched to pMDA, 66.8% (408/611) of individuals were eligible according to the inclusion and exclusion criteria and 91.9% (375/408) received pMDA. The follow-up CSS at 3 months after pMDA in round 2 was conducted among 1427 study participants, with 94.8% (579/611) in the pMDA clusters and 96.6% (848/878) in the control clusters. The additional postintervention CSS at 6 months after the second PQ administration was performed among 1401 study participants, with 92.9% (568/611) in the pMDA group and 94.9% (833/878) in the control group. Figure 3 presents the CONSORT (Consolidated Standards of Reporting Trials) diagram showing the flow of participants throughout the trial [29,30]. The CONSORT checklist is presented in Multimedia Appendix 1.

The demographic attributes and G6PD deficiency statuses of the study participants in the 7 clusters are shown in Tables 1 and 2. Based on the census data, there were slightly fewer male participants than female participants, with an age range of 1 to 96 years in both rounds of pMDA. Testing for G6PD deficiency revealed an overall prevalence of 5.4% (95% CI 4.3%-6.6%) in the 7 clusters. G6PD-deficient individuals were excluded from PQ administration.

**Table 1:** Baseline characteristics of the participants for round 1 of the primaquine mass drug administration and control clusters during the 2-period cluster-crossover sequence.

Variable	pMDA^a^ clusters					Control clusters						Total
	Cluster 1	Cluster 2	Cluster 3	Total of 1-3	Cluster 4		Cluster 5	Cluster 6	Cluster 7	Total of 4-7		
Pre-MDA^b^ CSS^c^, n	219	175	531	925	148		173	167	211	699	1624	
Male sex, n (%)	82 (37.4)	81 (46.3)	242 (45.6)	405 (43.8)	68 (45.9)		85 (49.1)	80 (47.9)	95 (45.0)	328 (46.9)	733 (45.1)	
Age (years), median (range)	32 (1-74)	29 (2-80)	40 (1-91)	37 (1-91)	29 (1-96)		31 (1-88)	26 (1-77)	46 (2-92)	34 (1-96)	35 (1-96)	
G6PD^d^ deficiency, n (%)	12 (5.5)	7 (4.0)	34 (6.4)	53 (5.7)	—^e^		—	—	—	—	—	

^a^pMDA: primaquine mass drug administration.

^b^MDA: mass drug administration.

^c^CSS: cross-sectional blood survey.

^d^G6PD: glucose-6-phosphate dehydrogenase.

^e^Not applicable.

**Table 2:** Baseline characteristics of the participants for round 2 of the primaquine mass drug administration and control clusters during the 2-period cluster-crossover sequence.

Variable	Control clusters					pMDA^a^ clusters						Total
	Cluster 1	Cluster 2	Cluster 3	Total of 1-3	Cluster 4		Cluster 5	Cluster 6	Cluster 7	Total of 4-7		
Pre-MDA^b^ CSS^c^, n	226	189	463	878	124		151	159	177	611	1489	
Male sex, n (%)	88 (38.9)	88 (46.6)	204 (44.1)	380 (43.3)	57 (46.0)		68 (45.0)	76 (47.8)	75 (42.4)	276 (45.2)	656 (44.1)	
Age (years), median (range)	32 (3-75)	28 (1-81)	42 (2-92)	36 (1-92)	36 (2-79)		31 (1-89)	27 (2-78)	48 (3-93)	36 (1-93)	36 (1-93)	
G6PD^d^ deficiency, n (%)	—^e^	—	—	—	8 (6.5)		4 (2.6)	9 (5.7)	13 (7.4)	34 (5.6)	—	

^a^pMDA: primaquine mass drug administration.

^b^MDA: mass drug administration.

^c^CSS: cross-sectional blood survey.

^d^G6PD: glucose-6-phosphate dehydrogenase.

^e^Not applicable.

### Potential Effectiveness of pMDA

To explore the potential effectiveness of pMDA, we compared the prevalences of *P. vivax* infections between the 2 study groups in each CSS period and the combined 2-period crossover. With the very low number of cases found for each CSS period, the prevalences were reported as the proportions of confirmed *P. vivax* infections per 1000 population. Among the CSS population in the pre-pMDA period of round 1 (July 2019), the proportions of *P. vivax* infections in the 2 groups were similar at around 14-15 per 1000 population. Among the CSS population in the 3-month post-pMDA period (November 2019), the proportions in the pMDA and control clusters reduced to 12.9 and 8.7 per 1000 population, respectively. Comparisons of the *P. vivax* proportions per 1000 population between the 2 groups (pMDA vs control) at each time period of round 1 showed no statistically significant differences (Table 3).

Among the CSS population in the pre-pMDA period of round 2 (July 2020), the proportions of *P. vivax* infections in the cross-over pMDA (clusters 4-7) and control (clusters 1-3) clusters were slightly different at 11.5 and 9.1 per 1000 population, respectively. Among the CSS population in the 3-month post-pMDA period (November 2020), the proportions in the pMDA and control clusters reduced to approximately 1.7 and 3.5 per 1000 population, respectively. Similarly, no statistically significant differences were found between the 2 groups in the round 2 study (Table 4). During the postintervention follow-up period, at 6 months after round 2 (March 2021), the proportions of *P. vivax* infections in the pMDA group (clusters 1-3) and control group (clusters 4-7) were 2.4 and 1.8 per 1000 population. Comparisons between the 2 groups also showed no significant differences (Table 4).

The inclusive effectiveness of pMDA for the 2-period crossover is shown in Table 5. In the pre-pMDA period, the inclusive numbers of participants across the 7 clusters (clusters 1-7), who received the pMDA intervention and control intervention, were 1536 (round 1 + round 2: 925+611) and 1577 (878+699), respectively. The proportions of *P. vivax* infections per 1000 population in the pMDA and control groups before pMDA were slightly different at 13.0 and 12.0 per 1000 population, respectively, with no statistically significant difference. In the 3-month post-pMDA period, the inclusive numbers of participants across the 7 clusters, who underwent the pMDA intervention and the control intervention, were 1430 (round 1 + round 2: 851+579) and 1420 (848+572), respectively. The proportions of *P. vivax* infections per 1000 population in the pMDA and control groups at 3 months after pMDA were 8.4 and 5.6, respectively, with no statistically significant difference.

**Table 3:** Comparisons of the proportions of *P. vivax* infections for round 1 of the primaquine mass drug administration and control clusters during the 2-period cluster-crossover sequence.

Variable	pMDA^a^ clusters				Control clusters						PR^b^ (95% CI)		*P* value
	Cluster 1	Cluster 2	Cluster 3	Total of 1-3^c^	Cluster 4	Cluster 5	Cluster 6	Cluster 7	Total of 4-7^c^				
Pre-pMDA CSS^d^, n	219	175	531	925	148	173	167	211	699	—^e^		—	
Pre-pMDA, n (infections/1000 population)	2 (9.1)	8 (45.7)	3 (5.7)	13 (14.1, 7.8-24.6)	0 (0.0)	8 (46.2)	3 (18.0)	0 (0.0)	11 (15.7, 8.3-28.9)	2.0 (0.1-34.3)		.55	
3 months post-pMDA CSS, n	206	163	482	851	108	148	155	161	572	—		—	
3 months post-pMDA, n (infections/1000 population)	1 (4.9)	7 (42.9)	3 (6.2)	11 (12.9, 6.8-23.7)	1 (9.3)	4 (27.0)	0 (0.0)	0 (0.0)	5 (8.7, 3.2-21.5)	2.3 (0.2-29.2)		.44	

^a^pMDA: primaquine mass drug administration.

^b^PR: prevalence ratio of the pMDA vs control groups based on a zero-inflated negative binomial mixed model (adjusted for cluster, without time period and interaction).

^c^For the total, the value of infections/1000 population is provided along with the 95% CI.

^d^CSS: cross-sectional blood survey.

^e^Not applicable.

**Table 4:** Comparisons of the proportions of *P. vivax* infections for round 2 of the primaquine mass drug administration and control clusters during the 2-period cluster-crossover sequence.

Variable	Control clusters				pMDA^a^ clusters						PR^b^ (95% CI)		*P* value
	Cluster 1	Cluster 2	Cluster 3	Total of 1-3^c^	Cluster 4	Cluster 5	Cluster 6	Cluster 7	Total of 4-7^c^				
Pre-pMDA CSS^d^, n	226	189	463	878	124	151	159	177	611	—^e^		—	
Pre-pMDA, n (infections/1000 population)	1 (4.4)	3 (15.9)	4 (8.6)	8 (9.1, 4.2-18.6)	0 (0.0)	7 (46.4)	0 (0.0)	0 (0.0)	7 (11.5, 5.0-24.5)	0.3 (0.0-8.1)		.38	
3 months post-pMDA CSS, n	226	188	434	848	108	148	163	160	579	—		—	
3 months post-pMDA, n (infections/1000 population)	0 (0.0)	1 (5.3)	2 (4.6)	3 (3.5, 0.9-11.2)	1 (9.3)	0 (0.0)	0 (0.0)	0 (0.0)	1 (1.7, 0.1-11.1)	0.5 (0.1-3.9)		.42	
6 months post-pMDA CSS, n	219	191	423	833	112	130	166	160	568	—		—	
6 months post-pMDA, n (infections/1000 population)	0 (0.0)	0 (0.0)	2 (4.7)	2 (2.4, 0.4-9.6)	0 (0.0)	1 (7.7)	0 (0.0)	0 (0.0)	1 (1.8, 0.1-11.4)	1 (0.0-54.2)		>.99	

^a^pMDA: primaquine mass drug administration.

^b^PR: prevalence ratio of the pMDA vs control groups based on a zero-inflated negative binomial mixed model (adjusted for cluster, without time period and interaction).

^c^For the total, the value of infections/1000 population is provided along with the 95% CI.

^d^CSS: cross-sectional blood survey.

^e^Not applicable.

**Table 5:** Comparisons of the proportions of *P. vivax* infections before and after primaquine mass drug administration between the intervention and control groups combined over the 2-period cluster-crossover sequence.

Variable	Intervention group (clusters 1-7)	Control group (clusters 1-7)	PR^a^ (95% CI)	*P* value
Pre-pMDA^b^ CSS^c^, n	1536	1577	—^d^	—
Pre-pMDA, n (infections/1000 population, 95% CI)	20 (13.0, 8.2-20.4)	19 (12.0, 7.5-19.1)	2.8 (0.3-28.4)	.37
3 months post-pMDA CSS, n	1430	1420	—	—
3 months post-pMDA, n (infections/1000 population, 95% CI)	12 (8.4, 4.6-15.1)	8 (5.6, 2.6-11.5)	2.1 (0.3-13.7)	.46

^a^PR: prevalence ratio of the pMDA vs control groups based on a zero-inflated negative binomial mixed model (adjusted for cluster, with time period as a covariate and interaction with the pMDA intervention).

^b^pMDA: primaquine mass drug administration.

^c^CSS: cross-sectional blood survey.

^d^Not applicable.

### Safety and Adverse Drug Reactions of PQ During pMDA

Adverse events potentially due to PQ intake were recorded in approximately 5% of participants in each round (30/575, 5.2% in round 1 and 20/375, 5.3% in round 2) (Table 6). Although study participants with G6PD deficiency were excluded, about 1% of the participants who received PQ showed symptoms suggesting acute hemolysis. Six participants had hemoglobin levels of <8 g/dL, and another 4 participants had dark urine. Major chief complaints after taking PQ varied and included headache, weakness, muscle ache and pain, and dry throat. All study participants with safety concerns were stopped from further PQ intake.

**Table 6:** Safety and adverse drug reactions during the 2-period cluster-crossover sequence.

Variable					Round 1 (n=575^a^), n (%)			Round 2 (n=375^a^), n (%)
**Total population with adverse effects**					30 (5.2)			20 (5.3)
	Hemoglobin level <8 g/dL			2 (0.3)			4 (1.1)	
	Dark urine			2 (0.3)			2 (0.5)	
	**Other adverse events (chief complaints)**							
		Headache	8 (1.4)			5 (1.3)		
		Weakness	7 (1.2)			1 (0.3)		
		Dry throat	5 (0.9)			0 (0.0)		
		Muscle ache and pain	1 (0.1)			4 (1.1)		
		Tachycardia	2 (0.3)			0 (0.0)		
		Chest tightness	1 (0.1)			2 (0.5)		
		Constipation	2 (0.3)			0 (0.0)		
		Diarrhea	0 (0.0)			1 (0.3)		
		Vomiting	1 (0.1)			1 (0.3)		
		Itching	0 (0.0)			2 (0.5)		

^a^Total population with at least 1-day drug administration.

### Acceptability of the pMDA Program

Information was collected from community representatives, including 12 from 2 villages in Yala Province and 12 from 2 villages in Narathiwat Province. Key informants also included 11 VHVs in the selected villages (7 from Yala and 4 from Narathiwat) and 18 representatives of health care personnel who worked in the study areas and MOPH consultative experts. Among the 53 key informants, 34 were male and 19 were female, and their age ranged from 18 to 86 years. Some of the study participants were community and religious leaders. Most VHVs and their family members participated in the pMDA activities. In assessing the acceptance of the pMDA program and stakeholder engagement, 5 themes were explored, namely, perceptions, expectations, and engagement; factors influencing the decision to participate or not participate; factors influencing noncompletion of the 14-day regimen; perceived achievements, blockages, and solutions in pMDA; and challenges in pMDA implementation.

#### Perceptions, Expectations, and Engagement

Study participants informed us that they were willing to participate in pMDA because they recognized that malaria was a major problem around their residential areas. Many of them, particularly the older generation, had experienced malaria. All villagers who agreed to the CSS, but could not participate in pMDA due to the exclusion criteria, perceived the program’s benefits. On the other hand, all those who rejected pMDA stated that they or their families had never been infected with malaria, that they would seek treatment should they be infected, and that prevention was not necessary based on these factors. VHVs, who were key players in pMDA implementation, perceived the program’s benefits. Almost all of them indicated that it would not be a burden as they have to perform home visits and other activities in the villages as part of their routine job anyway. All local health care officers at the district and subdistrict levels noted that they expected the program to help reduce malaria cases in their areas. They said that they had no worries about program implementation, as they could easily collaborate with community leaders and VHVs.

There were many malaria cases in our province – in the top ranking in Thailand. After the pMDA project, the malaria cases were reduced to none in our village… Some VHVs from other villages asked us why the project only came to our village.

VHV

#### Factors Influencing the Decision to Participate or Not Participate

Many participants said that they took a decision after going to one of several community engagement meetings arranged by the research team, community and regional leaders, and VHVs. Being well-informed about drug safety and the G6PD deficiency survey, most participants felt safe participating in the CSS and pMDA program.

Both of us took the drug for 14 days without any side effects. We decided to participate after attending the community meeting. We had no worries about taking the drug and the blood draw because we both used to get malaria. If we have to take the drug once a year, we still want to do so. We will ask our children to do so next time.

pMDA-compliant husband and wife

In contrast, all VHVs and local health care staff indicated that most teenagers and small children did not participate in the program. With the drug problem (including illegal herbs, amphetamine, cocaine, and other drugs), particularly among teenagers, they feared that the CSS would reveal their drug status. Some mentioned that taking medication for 14 days was too long and that they might participate in a shorter regimen. Cultural beliefs also affected the idea of taking medication. One community leader stated that some local people believed that eating durian (a local fruit) with malaria drugs might affect people’s health.

Two of us in the family did not participate even though we used to get malaria – this is because we had to go work in the forest and had no time for DOT. … My son aged 18 years old did not participate in the project because he had to go and study out of town…

pMDA nonparticipant

#### Factors Influencing Noncompletion of the 14-Day Regimen

All pMDA participants who did not complete the 14-day regimen indicated that they had to stop taking PQ because they had adverse drug reactions. A few had to withdraw via the VHVs owing to safety issues (eg, dark urine and hemoglobin <8 g/dL).

There are 5 of us in the family – only 3 participated in drug administration, the other 2 did not because of their pregnancies. I took the drug for only 4 days and stopped because I had a headache – if not, I would take it for the whole 14-days.

pMDA noncompliant person

#### Perceived Achievements, Blockages, and Solutions in pMDA

The brainstorming meeting reached a consensus that pMDA had an important impact on identifying asymptomatic infections because routine qPCR was not performed at local sites. The screening test for G6PD deficiency also contributed to significant implications, as there was little knowledge about G6PD deficiency prevalence in this region. Knowing the G6PD deficiency status instilled confidence in the local staff for PQ delivery during pMDA.

When asked about pMDA activities that required improvement, government officers recognized that community engagement still did not reach all target groups, and this was confirmed by villagers and VHVs. All stakeholders suggested that there should be greater coverage and more frequent community sensitization and engagement, which should be specific to the target groups (nonparticipant populations). Though willing to perform home visits, some VHVs noted that there should be some ways to handle intensive 14-day DOT. The brainstorming session also indicated a lack of resources (both workforce and financial support) that could obstruct the success of prevention and control measures.

At the beginning, we felt worried about our skills and the heavy workload of performing 14-day follow-ups. We also worried about getting villagers to understand the project, about taking the drug and the blood draw… The situation was better when we worked together with community leaders.

Local health care officer

#### Challenges in pMDA Implementation

As noted during the brainstorming session, human resources would be a major challenge when moving forward and upscaling pMDA. A few health authorities mentioned that in order to have a successful program, it must be a top-down approach, which means the policy must be initiated by authoritative bodies at higher levels and delegated to local operational entities. On the other hand, another MOPH authority noted that the local level should initiate the idea and propose it to the upper level. There were concerns about not only the human workforce but also budget allocation.

Taking a 14-day regimen requires a G6PDd screening test. This test is rather expensive – but if you do not do it, the villagers may not want to take the drug. This is important and we need to communicate well with villagers to have them take the drug. Importantly, we also need the full endorsement of higher-level authorities, i.e., MOPH. If the MOPH had such a policy, the local offices would do it.

MOPH authority at the local level

## Discussion

### Overview

The main goals of this study were to evaluate the effectiveness, safety, acceptability, and stakeholder engagement of pMDA to accelerate *P. vivax* elimination in Thailand and to provide the information needed by the Thai MOPH for evidence-based decision making. The study employed a mixed-methods approach. The quantitative part of the study employed a 2-period cluster-crossover randomized trial design assessing pMDA in addition to standard prevention and control measures. The qualitative part was performed by IDIs and brainstorming discussions.

### Effectiveness and Safety of the pMDA Intervention

As a proof-of-concept study, the potential effectiveness of the pMDA intervention was assessed by comparing the proportions of *P. vivax* infections between clusters under pMDA and clusters under SPC in each period and after the 2-period crossover. The study results indicated no statistically significant differences between the 2 groups both before and after pMDA implementation. However, there were reducing trends in *P. vivax* prevalence after pMDA implementation in both the treatment and control groups. In a systematic review in the Cochrane database, it was found that studies on MDA in Cambodia, Laos, Myanmar, and Vietnam in very low– to low-endemicity settings showed varying degrees of reductions in *P. vivax* prevalence immediately following the intervention, but the effects were not sustained [31]. Similarly, WHO guidelines for malaria in 2023 noted that MDA for *P. vivax* conducted in 8 countries showed rapid reductions in transmission with immediate- to short-term benefits 1-3 months after the last round of MDA, but long-term benefits at 12-24 months were not apparent [32]. In this study, the comparable reduction trends in the pMDA and control groups may reflect that pMDA was not as effective as anticipated. This might be due to the Hawthorne effect (the alteration of behavior by the study subjects due to their awareness of being observed), since both study participants and health care workers in the study areas were aware of the additional pMDA activities in their vicinity. It is also important to note that since the start of the study in 2019, drastically decreasing incidences have been observed across the country and not only in the study clusters. Moreover, people’s mobility was limited and malaria risk behaviors were less frequent during the COVID-19 pandemic, which coincidently started in 2019. Thus, it is difficult to reach definitive conclusions about the effectiveness of the pMDA intervention.

Consistent with the findings in other studies [15,20,33], pMDA was safe and well-tolerated. Testing for G6PD deficiency at the point of care before PQ administration is a precondition for safe administration [19]. Several previous studies noted that the tolerability of PQ has been good, with a low frequency of adverse events reported even with heterogeneous levels of G6PD deficiency [15,18]. This study, however, excluded individuals with inborn G6PD deficiency. As a safety monitoring measure, VHVs performed the intensive 14-day DOT of PQ receivers residing in households for which they were responsible. Other studies also reported that pMDA under supervision with good monitoring mechanisms for adverse events in the population would result in less severe adverse events related to PQ [15,18,19]. This study and other pMDA studies involving dihydroartemisinin-piperaquine and PQ showed similar common adverse events, including gastrointestinal disturbances (diarrhea, vomiting, abdominal pain, and nausea), dizziness, headache, and general body weakness [18,20,34]. Severe adverse events suggesting acute hemolysis occurred in 1.1% of PQ receivers, resulting in treatment cessation.

### Acceptability and Stakeholder Engagement in pMDA Activities

In the WHO manual for antimalarial MDA implementation, high coverage and adherence of the target population (ie, >80%) must be ensured, preferably implementing centralized distribution at a fixed site and performing DOT. In this study, only about 60% of participants consented to the initial CSS, but over 90% of them were retained in the 3-year study. Among those, only 70% were eligible for MDA, with 90% taking PQ and 90% completing the 14-day regimen under DOT. The qualitative part of this study suggested that acceptance of pMDA among study participants was predominantly due to their trust in health care representatives (VHVs) who actively performed home visits to provide health care support. This study confirmed that DOT, although labor-intensive, could maintain full adherence and reassure study participants about the purposes and safety of the MDA activities. pMDA and DOT heightened the morale and relationships between villagers and health care personnel in the study areas. Similarly, another study on reactive drug administration for *P. vivax* elimination in Thailand suggested that good acceptance of the program was related to education and sensitization campaigns on the purpose and rationale of the intervention [35]. Moreover, the WHO guidelines 2023 noted that a systematic review of 18 studies reported that the most common barrier to the acceptability of MDA for *P. vivax* was fear of adverse events, and some studies mentioned that sensitization on the benefits of MDA helped reduce concerns about adverse effects [32].

Community engagement is critical to the success of MDA for *P. vivax* infection as it affects participation rates and full treatment compliance, while lack of engagement with local health care providers limits treatment adherence [32]. A systematic review of published, unpublished, and gray literature documenting past MDA experiences identified the importance of operational implementation and community engagement, with drug distribution and DOT being mainly performed by community volunteers and local health workers [36]. A review of previous studies noted in the WHO guidelines 2023 also reflects that the impact of MDA on *P. vivax* infection, whether positive or negative, is likely related to the level of acceptance of the intervention among the malaria program staff, and there have been no surveys of these key stakeholders regarding this issue [32]. As suggested in the literature, all aspects of community engagement in MDA must be tailored to local (social, cultural, and political) circumstances [37,38]. This study involved community members as part of the intervention team and considered their customs and opinions. Study participants and public health care providers at local and national levels willingly accepted and believed that the preventive and control activities in this study (regardless of being in the pMDA or control groups) had boosted awareness in the community and led to personal changes in malaria-preventive behaviors. The qualitative part of this study confirmed the key determinants of pMDA, and it was shown that the feasibility of maintaining or upscaling the MDA intervention was related to the existence of active and continuous activities for community engagement, community sensitization, and maintaining collaborations with those from the point-of-care level up to authorities at local and national levels.

### Limitations of the Study

This study had several limitations. The number and size of clusters were small, and the study may not have observed heterogeneities among different groups. Limited mobility due to the COVID-19 pandemic coinciding with pMDA activities during the study follow-up period might have caused the malaria prevalence in all study clusters to decline drastically. Such circumstances complicate and hinder the interpretation of the results. Moreover, there were significant heterogeneities among the different clusters, making comparisons between the pMDA and control groups difficult. Regarding the study design, this study employed a cluster-crossover randomized trial design, which is particularly feasible for pragmatic clinical trial in health care systems [21,22]. The design is highly efficient and should be considered as it breaks up the total trial duration into a series of repeated measures. Thus, the required number of clusters could be substantially reduced while potentially providing generalizable, robust, and internally valid evidence for the evaluation of effects across settings or clusters [21,39,40]. Although the cluster-crossover design is robust when the number of clusters is small [26], this study involved only 7 clusters and the crossover sample size was around 1500 per group. Given the predictably large variation in malaria prevalence within each cluster, a larger sample size would have been needed. Thus, the results of this study may not have external validity and may not be generalizable to other settings with different natures of target populations and degrees of transmission.

### Conclusions

pMDA under DOT showed high adherence, safety, and tolerability, but it may not significantly impact *P. vivax* transmission, particularly in low-transmission areas. Although the impact of pMDA was inconclusive, the results were consistent. Malaria cases reduced in all clusters, regardless of whether they were in the pMDA group. The Hawthorne effect may reflect the trigger or accelerator of elimination by involving significant political, logistical, and financial commitments of coordinating bodies and collaborative efforts among various levels of stakeholders. We experienced success regarding effective health care workforces at point-of-care centers, effective collaborations in the community, and commitment from authorities at local and national levels. Such community engagement efforts boosted the acceptability of the malaria-elimination initiative.

Despite the safety and exemplary acceptance of the intervention, we cannot proclaim the effectiveness of pMDA. As we planned this study as a proof-of-concept study, the results were considered as a basis for decision making. A conditional recommendation in the WHO guidelines 2023 suggested the use of MDA for *P. vivax* infection when there is evidence of the acceptability, feasibility, impact (incidence or prevalence of malaria infection at the community level), and potential harm of MDA (including testing for G6PD deficiency) [32]. Since we did not observe the impact of pMDA on *P. vivax* transmission during the study period and the malaria incidence in Thailand increased in 2023 after the study period, we decided not to scale up the study with larger clusters and samples. Instead of implementing pMDA in the population within the intervention clusters, we are working on an on-going project with an alternative approach involving a targeted PQ treatment strategy that provides PQ via DOT only to the targeted population in households around each index case living in the intervention cluster. The effectiveness of such an alternative approach remains to be determined.

## Appendix

Multimedia Appendix 1 CONSORT (Consolidated Standards of Reporting Trials) checklist.

## Abbreviations

CONSORT: Consolidated Standards of Reporting Trials
CSS: cross-sectional blood survey
DOT: directly observed treatment
G6PD: glucose-6-phosphate dehydrogenase
GMS: Greater Mekong Subregion
ICEMR: International Center of Excellence for Malaria Research
IDI: in-depth interview
MDA: mass drug administration
MOPH: Ministry of Public Health
MPPT: mass primaquine preventive treatment
pMDA: primaquine mass drug administration
PQ: primaquine
SPC: standard of prevention and control
VHV: village health volunteer
WHO: World Health Organization

## Data Availability

All data and codes used in this analysis are presented in the manuscript.
